# Supplementary material for: Characterizing Aptamer Interaction with the Oncolytic Virus VV-GMCSF-Lact
Source: Molecules. 2024 Feb 14;29(4):848. doi: 10.3390/molecules29040848 (PMC10892425; doi:10.3390/molecules29040848)
Supplement: Supplementary file 1 [file molecules-29-00848-s001.zip › molecules-2807251-supplementary.pdf]

Supplementary Materials

Nuclease Resistance of aptamer

Renatured NV14t_56 were incubated in 10% human serum containing anti-vaccinia neutralizing antibodies (S1) in IMDM culture medium (Sigma, Burlington, MA, USA) for 2 h at 37C. After fixed times (in 30 seconds, 1 min, 2 min, 5 min, 10 min, 30 min, 1 hour, 2 hours), 10 pmol aliquots of this mixture were taken. Stop buffer (8 M urea, 0.01% bromophenol blue) was added to the aliquots and set at minus 20 degrees. The cleavage of aptamer by nuclease of serum was analyzed by gel electrophoresis in 15% denaturing polyacrylamide gel and visualized by fluorescence of Cy5 on 600 nm using the Amersham Typhoon fluorescent scanner FLA 9500 (Cytiva, Uppsala, Sweden). The images were quantified using the Quantity One program (Bio-Rad, USA). The percent of aptamer cleavage was calculated as reported in [1]. The obtained values were further processed by GraphPad Prism 6 (GraphPad Software, San Diego, CA, USA).

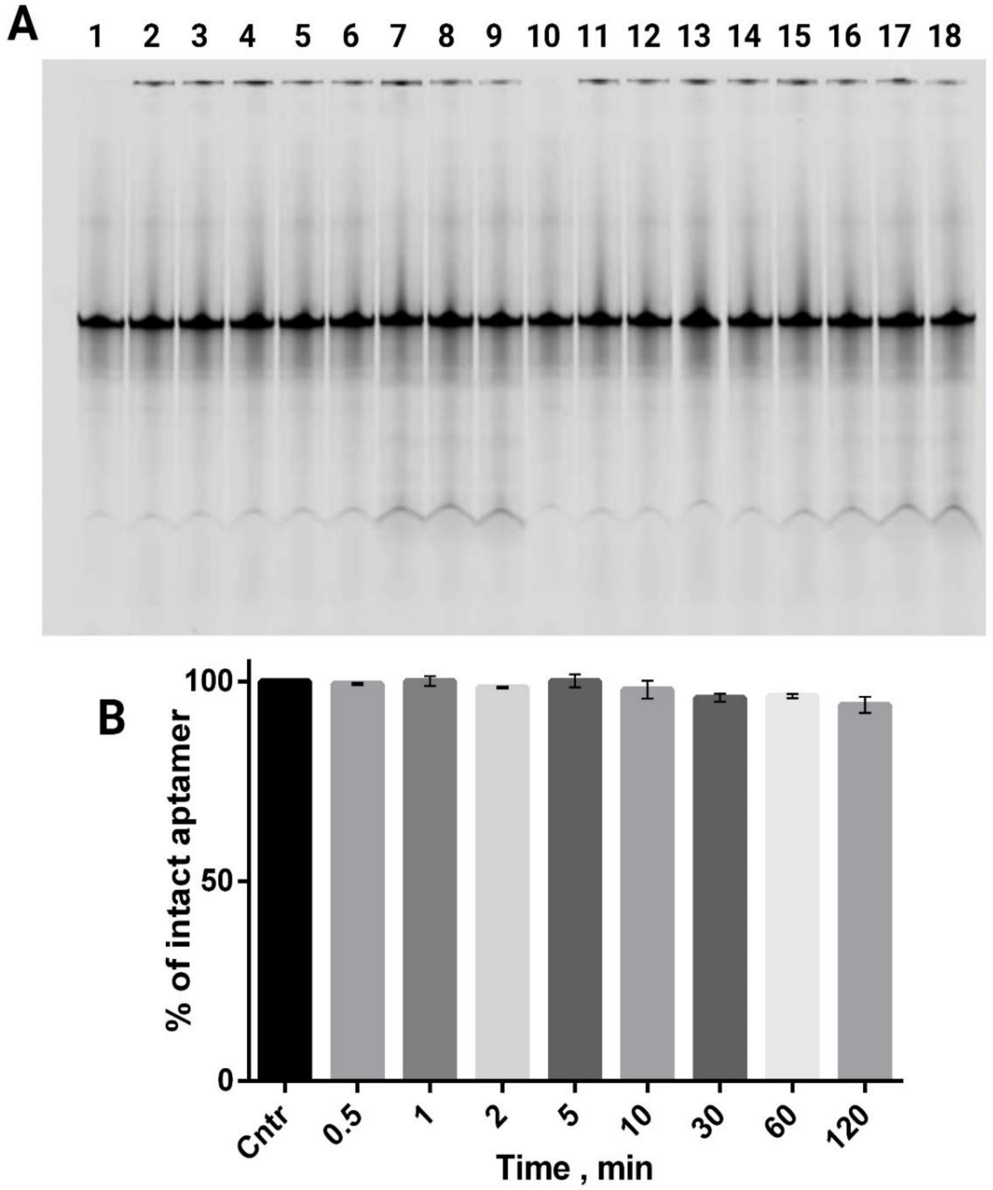

Figure S1. Stability of the aptamer NV14t_56 in the presence of human serum containing anti-vaccinia neutralizing antibodies (S1). Conditions: concentration of aptamer 10 pmol, 37^0C, 2 h.

A - Electrophoretic analysis of degradation products in a 15% denaturing polyacrylamide gel, bands: №№1,10 – Cntr (aptamer without serum), №№ 2, 11 – 0,5 min, №№ 3, 12 – 1 min, №№ 4, 13 – 2 min, №№ 5, 14 – 5 min, №№ 6, 15 – 10 min, №№ 7, 16 – 30 min, №№ 8, 17 – 60 min, №№ 9, 18 – 120 min. Cntr is a control sample containing only 10 pmol of aptamer. **B** - Plot of percentage of intact aptamer versus time in the presence of serum.

Using one –way ANOVA algorithm we found that there are statistical differences ($P \leq 0.05$) between following groups: Cntr vs 120 min, 0,5 min vs 120 min, 1 min vs 120 min, 5 min vs 120 min.

1. Sakovina, L.; Vokhtantsev, I.; Vorobyeva, M.; Vorobyev, P.; Novopashina, D. Improving Stability and Specificity of CRISPR/Cas9 System by Selective Modification of Guide RNAs with 2′-fluoro and Locked Nucleic Acid Nucleotides. *Int. J. Mol. Sci.* **2022**, *23*, 13460, https://doi.org/10.3390/ijms232113460.
